# Metamaterial Sensing of Cyanobacteria Using THz Thermal Curve Analysis

**DOI:** 10.3390/bios14110519

**Published:** 2024-10-23

**Authors:** Tae Hee Jeong, Seung Won Jun, Yeong Hwan Ahn

**Affiliations:** Department of Physics and Department of Energy Systems Research, Ajou University, Suwon 16499, Republic of Korea; hyjoun64@ajou.ac.kr (T.H.J.); sos123dssud@ajou.ac.kr (S.W.J.)

**Keywords:** terahertz, cyanobacteria, metamaterials

## Abstract

In this study, we perform thermal curve analyses based on terahertz (THz) metamaterials for the label-free sensing of cyanobacteria. In the presence of bacterial films, significant frequency shifts occur at the metamaterial resonance, but these shifts become saturated at a certain thickness owing to the limited sensing volume of the metamaterial. The saturation value was used to determine the dielectric constants of various cyanobacteria, which are crucial for dielectric sensing. For label-free identification, we performed thermal curve analysis of THz metamaterials coated with cyanobacteria. The resonant frequency of the cyanobacteria-coated metasensor changed with temperature. The differential thermal curves (DTC) obtained from temperature-dependent resonance exhibited peaks unique to individual cyanobacteria, which helped identify individual species. Interestingly, despite being classified as Gram negative, cyanobacteria exhibit DTC profiles similar to those of Gram-positive bacteria, likely due to their unique extracellular structures. DTC analysis can reveal unique characteristics of various cyanobacteria that are not easily accessible by conventional approaches.

## 1. Introduction

As a consequence of global warming and eutrophication, cyanobacterial blooms have become a major environmental problem associated with freshwater systems [1,2,3,4,5], causing cyanobacteria to grow more rapidly and aquatic ecosystems to collapse because of a reduction in dissolved oxygen levels [6,7,8,9]. To mitigate the adverse effects of the accelerated growth rate of cyanobacteria in aquatic ecosystems, it is essential to establish methods to identify and classify different species of cyanobacteria. Microscopic techniques and polymerase chain reactions (PCR) have been widely used to identify cyanobacteria [10,11,12,13,14,15]. However, microscopic techniques are limited to identifying bacteria based on shape and color, and it may be difficult to distinguish cyanobacteria of the same shape and color [16]. Comparatively, PCR techniques are accurate but time-consuming and require specific DNA primers [17,18,19,20]. Therefore, it is imperative to develop an accurate tool to identify cyanobacterial species.

Terahertz (THz) metamaterials have recently been introduced as effective platforms for real-time sensitive microorganism detection [21,22,23,24,25,26,27,28]. Since metamaterial sensing involves dielectric sensing, determining the optical index of the target substances is the first step in practical sensor applications [29,30,31,32]. As microorganisms can now be classified based on their dielectric properties, bacteria have been found to exhibit higher dielectric constants than molds, whereas yeasts exhibit higher values than water [33]. The differences in the dielectric properties can be attributed to differences in the cell wall composition. Furthermore, we developed a label-free THz spectroscopic method for identifying individual bacterial species based on temperature-dependent peak shifts of metamaterials [34]. The differential thermal curves (DTC) of the metamaterial resonance were obtained by monitoring the temperature-dependent frequency shift, which provides unique fingerprints of individual microbes. Therefore, a novel THz-sensing technique for identifying microbial species based on their intrinsic properties will help prevent cyanobacterial blooms.

In this study, we used a metamaterial sensor to determine the dielectric constant of cyanobacteria by measuring the shift in the metamaterial resonance of four types of cyanobacteria. In addition, we monitored the THz transmission spectra when the metasensors coated with cyanobacteria were heated. This allowed us to address temperature-dependent changes in the cyanobacterial dielectric constant, which provides unique fingerprints for the label-free identification of cyanobacteria.

## 2. Experimental Setup

**Figure 1:**
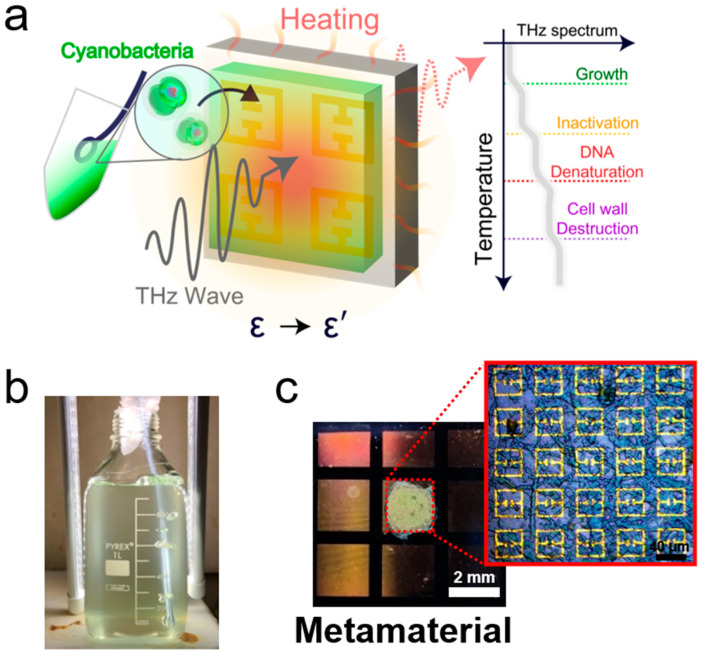
(**a**) Schematic illustration of the dielectric constant measurement with THz metamaterials and thermal curve analysis for label-free identification. (**b**) Picture of bottled water containing cyanobacteria (*Anabaena* sp.) grown by blue-green medium (BGII). (**c**) Photograph of metasensor coated with *Anabaena* sp.

We determined the dielectric constants of the cyanobacterial layers using metasensors and performed THz thermal curve analysis for label-free identification. As schematically illustrated in Figure 1a, we coated the cyanobacterial films on a metamaterial pattern and recorded the metamaterial resonance (*f*_R_) shift as a function of the sample temperature from 25 to 160 °C. In general, a bacterial cell experiences multiple phases as its temperature rises, such as growth, thermal inactivation, DNA denaturation, and wall destruction [35,36,37]. Accordingly, significant changes in the THz dielectric constant should occur at the transition temperature between the multiple growth and death phases in cyanobacteria. Individual microbial species have different temperature-dependent characteristics; therefore, we can identify metamaterial resonances by measuring them at different temperatures without pretreatments such as labeling and DNA extraction.

Cyanobacterial samples from the Korean Collection for Type Cultures (*Anabaena* sp. AG10059, *Aphanocapsa* sp. AG10016, and *Aphanothece* sp. AG10010) and Freshwater Bioresources Culture Collection (*Microcystis* sp. FBCC-A68) were grown in 1 L of DI water mixed with 1.64 g of blue-green medium (BGII). To promote cyanobacteria growth, the temperature was maintained at 20 °C (*Microcystis* sp.), 25 °C (*Anabaena* sp. and *Aphanothece* sp.), and 30 °C (*Aphanocapsa* sp.) using a hot plate. They were grown for more than two weeks under 5000 lx of light illumination for 9–12 h each day, whereas it took almost a month to grow some species (e.g., *Microcystis* sp. and *Aphanocapsa* sp.). Using a conventional photolithography technique, we fabricated THz metamaterials on silicon substrates (0.5 mm thick), followed by metal evaporation of Cr/Au (10 nm/90 nm). It consisted of a 40 × 40 array of split-ring electrical resonators with a side arm length of 36 μm, line width of 4 μm, gap width of 3 μm, and periodicity of 50 μm.

A conventional THz time-domain spectroscopy (THz-TDS) system was used to measure the transmission amplitudes of the THz metamaterial devices [21,22,23,24,25]. The linearly polarized THz pulse was generated by illuminating a photoconductive antenna (GaAs) with a femtosecond laser (centered at *λ* = 800 nm). By varying the time delay between the THz pulse and femtosecond probe beam, time traces of the transmitted THz electric field were obtained, and the THz spectrum could then be derived from these time traces using a fast Fourier transform. The in situ THz absorption of the metamaterials coated with cyanobacteria was monitored using ceramic heaters to adjust the sample temperature. The THz pulses were focused on a metamaterial with a focusing area of 1 mm^2^. The ceramic heater was punctured at its center with a diameter of 2 mm to facilitate the transmission experiments. The temperature of the sample was monitored by using a temperature sensor attached to the ceramic heater (and confirmed by a pyrometer) and we recorded the transmission spectrum as a function of the measured temperature. We increased the temperature gradually (from 25 to 160 °C for 40 min); the temperature was uniform over the sample surface.

**Figure 2:**
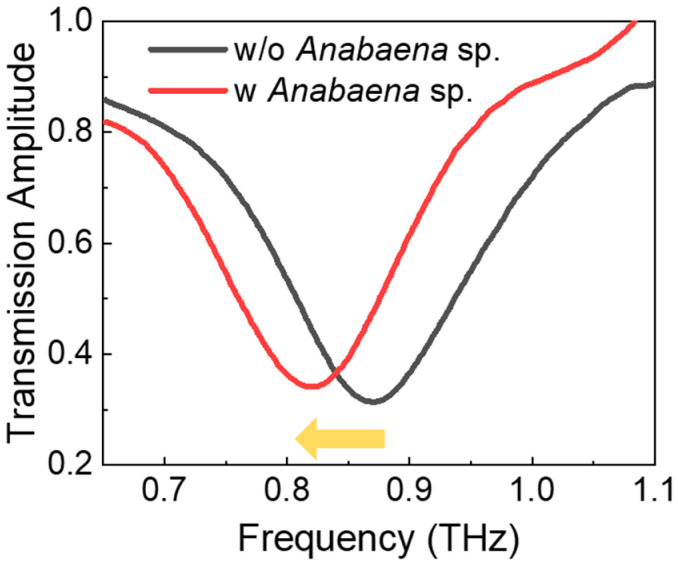
THz transmission amplitudes through the metamaterial with (red) and without (black) coating of the cyanobacteria (*Anabaena* sp.) The thickness of the films was about 4 μm.

We measured the dielectric constants of the cyanobacterial layers using the saturation thickness response of metamaterial resonance [38]. Information on the dielectric constant (ε_r_) of the target materials is crucial in dielectric sensing; however, these information have not been addressed for cyanobacteria in the THz frequency range. Metamaterial sensing has been proven to be an effective technique for obtaining the dielectric constants of polymer films and liquids. These methods are free from interference effects and do not require large sample quantities because the metamaterial sensing volume is quite confined near the gap structure. We measured the THz transmission amplitude of the metamaterial before and after deposition of the cyanobacterial layers, as shown in Figure 2. THz transmission spectra are shown with (red) and without (black) coating of the cyanobacterial film consisting of *Anabaena* sp. with a thickness of 4 μm. The cyanobacterial layer was deposited using the drop-casting method after rinsing the cyanobacterial solution by centrifugation at 12,000 rpm for 30 min (repeated three times). This process effectively removed the culture medium from the solution. We used metamaterials with a resonant frequency of 0.87 THz without coating of the cyanobacteria. Conversely, the resonance shift was measured at ~50 GHz when we coated *Anabaena* sp., as shown in Figure 2.

Several geometrical factors affect the resonant frequency (*LC* resonance frequency) of THz metamaterials, including the gap width, sidearm length, and refractive index of the substrate (*n*_sub_) [23,39,40]. A critical aspect of *LC* resonance is that it is influenced by the dielectric environment of the metamaterial; in other words, the resonant frequency of the metamaterial is inversely related to the effective refractive index *n*_eff_. Here, *n*_eff_ is a linear combination of the refractive indices of the substrate and air refractive indices [41]. In metamaterial sensing, additional dielectric materials (such as cyanobacteria) change the effective dielectric constant in the gap areas of the metamaterial, causing a redshift in the THz transmission spectrum. As a result, the resonance shift (Δ*f*) can be expressed by the following relationship: Δ*f*/*f*_0_ ≈ *α*(*ε*_r_ − *ε*_air_)/*ε*_eff_ [42]_,_ where *α* is the sensitivity coefficient, *ε*_air_ is the dielectric constant of air, and *ε*_eff_ (=*n*_eff_^2^) is the effective dielectric constant without the dielectric film coating.

**Figure 3:**
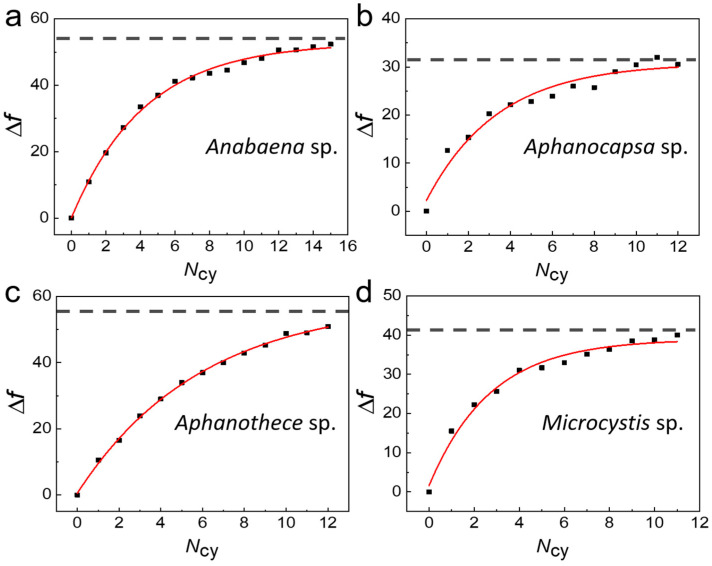
Resonant frequency shift (Δ*f*) as a function of coating time (*N*_cy_) for four different cyanobacterial species: *Anabaena* sp. (**a**), *Aphanocapsa* sp. (**b**), *Aphanothece* sp. (**c**), and *Microcystis* sp. (**d**). Red solid lines indicate fits to the data.

Figure 3a shows a plot of Δ*f* as a function of deposition time (*N*_cy_) of cyanobacteria films for *Anabaena* sp. We measured the changes in the THz spectrum transmitted through microgap metamaterials after the deposition of four different cyanobacteria prevalent in Korean rivers. The cyanobacteria were drop-cast from the solution at a density of 18 mg/mL. In each deposition, we used 5 μL solution to fill the area by using a polydimethylsiloxane (PDMS) well with a 2 mm diameter, followed by a drying process under ambient conditions. As we increased *N*_cy_ (i.e., with increasing layer thickness), Δ*f* increased due to the change in the effective dielectric constant of the gap area. For dielectric constant measurements, we removed the culture medium completely, because it distorts dielectric values significantly. For that purpose, the cyanobacterial layer was deposited using the drop-casting method after rinsing the cyanobacterial solution by centrifugation at 12,000 rpm for 30 min (repeated three times). Δ*f* was saturated at a specific thickness with a deposition time of *N*_sat_ because the effective sensing volume of the THz-metamaterial sensor is highly confined near the surface. We extracted the saturation value Δ*f*_sat_ using the following fittings: $\Delta f=\Delta f_{\mathrm{sat}}(1-\exp(-N_{\mathrm{film}}/N_{\mathrm{sat}}))$ [22,23,39]. Δ*f*_sat_ of 54.9 GHz (black dashed line) was obtained, as shown in Figure 3a. We also performed similar experiments on other cyanobacterial species, as shown in Figure 3b–d, respectively, for *Aphanocapsa* sp., *Aphanothece* sp., and *Microcystis* sp. We used the same experimental conditions as for the *Anabaena* sp. case, including the solution density; however, the thicknesses varied depending on their individual sizes. The saturated frequency shifts were measured to be 31.3 GHz (*Aphanocapsa* sp.), 58.4 GHz (*Aphanothece* sp.), and 41.6 GHz (*Microcystis* sp.).

**Figure 4:**
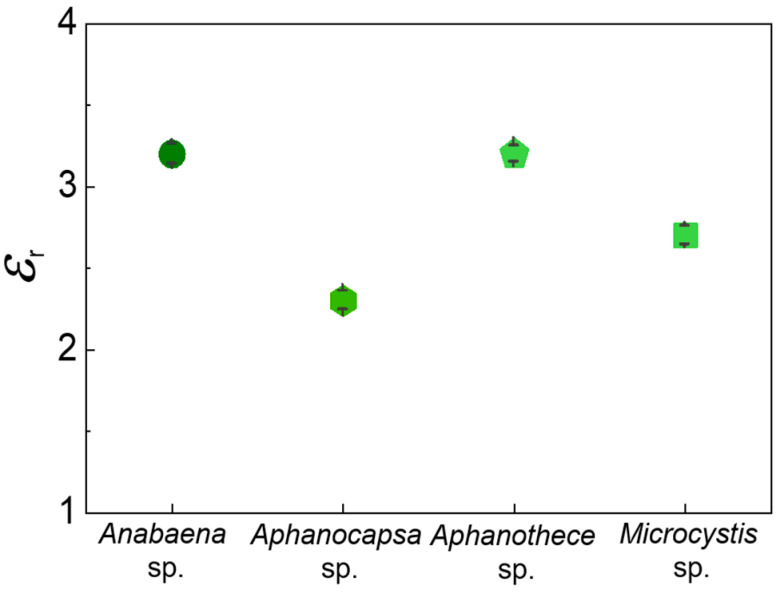
Dielectric constant measurement results for different cyanobacterial layers. The error bars indicate the standard deviation from the fitting.

We determined the dielectric constant of cyanobacteria films, as shown in Figure 4, because it has a close correlation to Δ*f*_sat_/*f*_0_ values. In our previous studies [21], we found an explicit correlation between the two quantities when the film was sufficiently larger than the saturated thickness. THz metamaterial sensors can be used to measure the dielectric constant of target materials without determining the precise thickness. Based on the relationship between *ε*_r_ and Δ*f*_sat_/*f*_0_ for metamaterial devices used in this study: *ε*_r_ = 33.4·(Δ*f*/*f*_0_) + 0.99 [38], the dielectric constants of cyanobacteria films were found from Δ*f*_sat_/*f*_0_ to be 3.2 (*Anabaena* sp.), 2.3 (*Aphanocapsa* sp.), 3.2 (*Aphanothece* sp.), and 2.7 (*Microcystis* sp.). The results are summarized in Figure 4, addressing the dielectric information of cyanobacteria in the THz frequency range. Our results are consistent with the dielectric indexes of other bacterial films (2.0–2.7) reported previously [33], whereas the values for *Anabaena* sp. and *Aphanothece* sp. are relatively higher. Dielectric information is the first step in metamaterial sensing applications. If the target amount is given, careful measurement of the metamaterial resonant frequency shift would be useful in identifying individual cyanobacteria. In particular, it is useful for early identification of pathogen types because they can be classified in terms of their dielectric values [33]. Conversely, thermal curve analysis will provide a unique approach to label-free identification without knowing the amount of the target materials [34].

**Figure 5:**
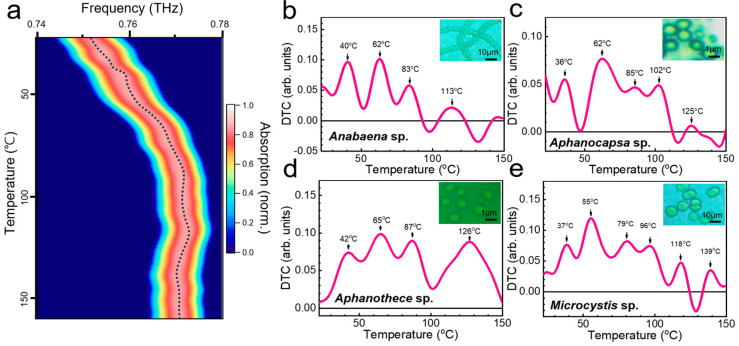
(**a**) 2D plot of THz absorption through metamaterials coated with cyanobacteria (*Anabaena* sp.) as functions of spectrum (*x*-axis) and substrate temperature (*y*-axis). (dotted line) Metamaterial resonance (*f*_R_) as a function of temperature. (**b**) Differential thermal curves (d*f*_R_/d*T*) for *Anabaena* sp. layer obtained by differentiating the curve in (**a**). (**c**–**e**) Differential thermal curves *Aphanocapsa* sp., *Aphanothece* sp., and *Microcystis* sp. layers.

We obtained differential thermal curves, revealing multiple peaks at the transition temperature, which enabled us to identify cyanobacteria [34]. Figure 5 shows a representative result of DTC analysis based on in-situ THz spectroscopy, in which the temperature of the metasensors coated with cyanobacterial layers was increased. Figure 5a illustrates a two-dimensional (2D) plot of THz absorption versus temperature when the metasensor is covered with a 12 μm-thick *Anabaena* sp. layer. The sample temperature was gradually increased from 25 to 160 °C for 40 min. The initial peak frequency was 0.75 THz at room temperature, which corresponds to the resonant frequency of the metasensor when covered by *Anabaena* sp. layer. In contrast, *f*_R_ increased as the dielectric constant of the coated film decreased with increasing temperature. We note that the film contained the culture media (BGII) used for growth. However, the presence of the culture medium did not significantly influence the DTC curves. When they are covered by culture media without cyanobacteria, the metamaterial resonance does not change with the temperature of our interest.

As the temperature increased, *f*_R_ exhibited a blue shift until the temperature reached 100 °C, indicating that the dielectric constant of the cyanobacterial layer decreased as the temperature increased. In addition, the metamaterial resonance did not change significantly without microbial films beyond the temperature range of interest. By fitting the curve in Figure 5a, we plotted *f*_R_ as a function of temperature *T*, as illustrated by a dotted line. Finally, the change in the resonant frequency at the transition stages was better illustrated using the differential thermal curve based on d*f*_R_/d*T* (Figure 5b). Peaks were observed at 40, 62, 83, and 113 °C. As the temperature increased, the dielectric constant decreased (which causes the metasensor resonance to shift blue). This can be attributed to cell expansion and molecular structural changes that occur during cell growth and death. For instance, it is well known that at growth temperatures, microbes undergo proteolysis, during which they break down proteins into their component amino acids [37,43,44,45,46]. In contrast, thermal inactivation occurs primarily because of protein denaturation, and a decrease in the DC dielectric constant has been previously reported at high temperatures [47]. The denaturation of DNA reduces its dielectric index [48]; however, the detailed characteristics of DNA in the THz range remain to be determined. DTC curves for other cyanobacteria are shown for *Aphanocapsa* sp. (Figure 5c), *Aphanothece* sp. (Figure 5d), and *Microcystis* sp. (Figure 5e) layers, which shows different characteristics relative to *Anabaena* sp. layer. The unique temperature-dependent changes in the dielectric indices of microorganisms allow THz thermal curves to provide unique fingerprints for the identification of cyanobacteria.

**Figure 6:**
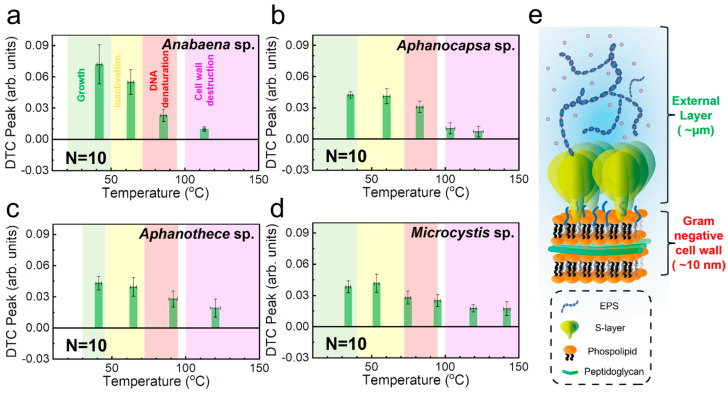
Bar graphs of amplitude as a function of temperature according to the peaks observed in DTCs for *Anabaena* sp. (**a**), *Aphanocapsa* sp. (**b**), *Aphanothece* sp. (**c**), and *Microcystis* sp. (**d**). The error bars indicate the standard deviation obtained from statistics for DTC curves from 10 samples of each species. Background colors indicate temperature ranges for their growth (green), thermal inactivation (yellow), DNA denaturation (red), and cell wall destruction (purple) reported in the literature. (**e**) Schematic illustration of extracellular structures in cyanobacteria.

Figure 6 summarizes the DTC analysis results for the four cyanobacterial species in terms of peak amplitude vs. temperature. For each species, we averaged the results of ten separate DTC measurements. All the samples exhibited multiple peaks that could serve as potential fingerprints for identification. The error bars represent the standard deviation of 10 samples each. The DTC peak positions are consistent with those reported in the literature, as indicated by the numbers in parentheses in Table 1. Specifically, these peaks fall within the temperature ranges for growth (shaded green in Figure 6), thermal inactivation (yellow), DNA denaturation (red), and cell-wall destruction (purple), thus validating our model. These measurements were performed using various biological techniques including optical density measurements, flask methods, PCR, and differential scanning calorimetry. However, conventional methods usually require a substantial number of samples or are time-consuming. Furthermore, although the dielectric constants of the bacteria are similar, we could distinguish them according to their species using the melting curve analysis obtained from the temperature-dependent dielectric constants. Interestingly, multiple peaks are found within death phases for some cyanobacteria species. For instance, double peaks were observed for DNA denaturation peaks for *Microcystis* sp. in Figure 6d; this is consistent with previous literature reporting two types of genes with different melting points [49,50]. Furthermore, the multiple peaks found in the cell wall detection phases in Figure 6b,d are also consistent with multiple polysaccharide components present in *Aphanocapsa* sp. and *Microcystis* sp. [51,52,53]. It is noteworthy that the peak positions in the melting curves can differ from our data depending on the growth conditions in the culture medium. Future research is required to address DTC curves based on growth conditions and layer thicknesses [37].

Notably, the DTC peaks of cyanobacteria revealed cell wall destruction (shaded purple in Figure 6), although cyanobacteria are classified as Gram negative [54]. Previous research has demonstrated that DTC analysis can classify bacteria by Gram type based on these cell wall destruction peaks, which are generally missing in Gram-negative bacteria [34]. The presence of cell wall destruction peaks for cyanobacteria can be attributed to the presence of external layers on the outer surface of cyanobacteria (as illustrated in Figure 6e), which consist of extracellular polymeric substances (EPS) and mucilaginous sheaths [55]. In addition, some of cyanobacteria (for *Aphanocapsa*, *Aphanothece*, and *Microcystis* species) contain S-layers that are not found in other bacteria [56,57]. The thickness of these additional structures could reach a couple of micrometers [58], much thicker than the cyanobacterial cell wall itself. In particular, the EPS contains a substantial amount of peptidoglycan, which is the basic component of bacterial cell walls. This abundance of peptidoglycan within the EPS significantly affects the optical dielectric constant observed in the DTC profiles. Consequently, their dielectric properties resemble Gram-positive bacteria. It is also known that the EPS dissolve at temperatures greater than 100 °C [55,59], which is consistent with our DTC results. The unique features of cyanobacteria are challenging to examine using conventional imaging systems [60]. Conversely, they are effectively captured through DTC analysis despite their classification as Gram-negative bacteria; therefore, DTC analysis is very useful for revealing and highlighting the structural characteristics of various microbial systems.

**Table 1:** Phase transition temperatures from DTC results for different cyanobacterial species.

Microorganism	Growth	Inactivation	DNA Denaturation	Cell Wall Destruction	Refs.
*Anabaena* sp.	42 °C (20–50 °C)	64 °C (>50 °C)	86 °C (72–94 °C)	114 °C (>100 °C)	[61,62,63,64,65,66,67]
*Aphanocapsa* sp.	36 °C (15–40 °C)	60 °C (>40 °C)	83 °C (72–94 °C)	104, 123 °C (>100 °C)	[61,62,66,67,68,69]
*Aphanothece* sp.	41 °C (30–45 °C)	65 °C (>45 °C)	92 °C (72–94 °C)	121 °C (>100 °C)	[61,62,66,67,70]
*Microcystis* sp.	34 °C (10–40 °C)	54 °C (>40 °C)	75, 95 °C (72–94 °C)	119, 142 °C (>100 °C)	[61,62,66,67,71,72,73]

## 3. Conclusions

In this study, we demonstrated label-free cyanobacterial sensing using THz-metamaterials. Upon depositing cyanobacterial layers on a metamaterial, the resonant frequency shifts but then saturates at a certain thickness owing to the limited sensing volume of the metamaterial. Based on the saturation values, we determined the dielectric constants of the four cyanobacteria commonly found in rivers. The dielectric index of the film ranges from 2.3 to 3.2, which is consistent with the bacterial values. For potential label-free identification, we performed thermal curve analysis using THz metamaterials on cyanobacteria. The resonant frequency of the metasensor coated with the cyanobacterial layers changed with temperature in accordance with their respective transition temperatures for growth, thermal inactivation, DNA denaturation, and cell wall destruction. DTC derived from the temperature-dependent resonance showed peaks unique to different cyanobacterial species, thereby enabling species identification. Interestingly, despite being classified as Gram-negative bacteria, cyanobacteria exhibit DTC peaks for cell wall destruction; these peaks are similar to those of Gram-positive bacteria and result from extracellular structures. These characteristics, as revealed by DTC analysis, underscore the complex and robust nature of cyanobacterial cell walls. Our results can be further extended to the study of other cyanobacterial species, showing potential for the development of highly sensitive onsite identification methods for various hazardous substances in rivers.

## Data Availability

The original contributions presented in the study are included in the article, further inquiries can be directed to the corresponding author.
